# Right Ventricular Outflow Tract and Pulmonary Artery Strains During the Cardiac Cycle Prior to Pulmonary Valve Replacement

**DOI:** 10.1007/s00246-025-04034-w

**Published:** 2025-09-30

**Authors:** Liam Swanson, Raphael Sivera, Nicholas M. Jacobson, Claudio Capelli, Lorna P. Browne, Catalina Vargas-Acevedo, Gareth J. Morgan, Silvia Schievano

**Affiliations:** 1 Institute of Cardiovascular Sciences, University College London, London, UK; 2 Great Ormond Street Hospital for Children NHS Foundation Trust, London, UK; 3 College of Engineering, Design and Computing, University of Colorado, Aurora, CO USA; 4 Department of Pediatric Radiology, University of Colorado School of Medicine, Aurora, CO USA; 5 Department of Pediatric Cardiology, University of Colorado, Aurora, CO USA; 6 The Heart Institute, Children’s Hospital Colorado, Aurora, CO USA; 7 School of Medicine, Anschutz Medical Campus, University of Colorado, Aurora, CO USA; 8 Department of Cardiology, University of Colorado Hospital, Aurora, CO USA

**Keywords:** Right ventricular outflow tract, Pulmonary artery, Pulmonary valve replacement, Strains, Deformable shape modeling

## Abstract

Right ventricular outflow tract and pulmonary arteries (RVOT/PAs) in surgically repaired congenital heart disease are highly dynamic structures, dilating in systole to accommodate the ejected blood volume while also elongating due to the ventricular contraction. In this study, we aim to quantify RVOT/PA circumferential and axial strains in a population of patients assessed for transcatheter pulmonary valve replacement (TPVR). 3D RVOT/PA geometries at end diastole and systole were reconstructed from 4D computed tomography images of 20 patients (35% female; 34 ± 15 years; 50% pulmonary stenosis, 40% Tetralogy of Fallot). A deformable shape model (DSM) was built to establish point-correspondence between subjects at end diastole and measure the RVOT/PA deformations of each subject to systole. The strain components were averaged over RVOT, pulmonary valve, and pulmonary trunk, and the strain ratio, describing the simultaneous deformations in the two main loading directions, was calculated. Overall, circumferential strains were smaller (range: − 0.28–0.30; area-weighted average 0.09), than axial strains (range: – 0.16–0.95; area-weighted average 0.28). Axial strains were high in the RVOT and progressively decreased toward the PA bifurcation, while circumferential strains increased instead. 90% of RVOT/PA showed positive strain ratios (0.13 ± 0.31, 0.25 ± 0.20, and 1.04 ± 0.52 in the RVOT, pulmonary valve, and pulmonary trunk, respectively). The RVOT/PA DSM computed in TPVR patients confirmed the macroscopic positive strain ratio observed during the cardiac cycle, with overall greater axial strains compared to circumferential strains. This may have implications in device/patient selection for TPVR.

## Introduction

Transcatheter pulmonary valve replacement (TPVR) offers a minimally invasive approach to pulmonary valve replacement following surgical repair of congenital heart disease (CHD) that affects the right ventricular outflow tract (RVOT), pulmonary valve, and pulmonary arteries (PAs), referred jointly as the RVOT/PA, such as Tetralogy of Fallot, pulmonary stenosis, and pulmonary atresia [1, 2]. The latest generation of TPVR device families have been designed for large, dynamic, and/or native RVOT/PAs [3–6], representing a leap forward in transcatheter approached to pulmonary valve replacement. Four-dimensional computed tomography (4DCT) is recommended for patient screening and TPVR device selection, to minimize risk of device migration and paravalvular leakage on one hand, and vessel rupture and stent failure on the other [7, 8]. In previous studies, 4D imaging showed that the global RVOT/PA dynamics during the cardiac cycle is large in the circumferential (i.e., dilatation) and axial (i.e., elongation) directions, as a result of the stroke volume pumped during systole that makes the RVOT/PA dilate [9, 10], and the ventricular contraction that forces RVOT/PA elongation. However, despite the increasing availability of new TPVR technologies on the market, current stent designs cannot accommodate for these simultaneous circumferential and axial deformations, as when expanded, they shorten, thus stiffening the RVOT/PA after implantation, especially in the axial direction. With the increased focus on CHD lifelong management, the inherent TPVR stent mechanical behavior may have consequences on the long-term loading of the right ventricle, altered pulmonary hemodynamics and increased fatigue on the stent.

Deformable shape modeling (DSM) methods have previously been used to uncover population complex shape characteristics in several areas of medical research including cardiovascular applications [11–14]. DSM ensures point-to-point correspondence between anatomical features that are otherwise difficult to select due to lack of clear anatomical landmarks [15–17]. Combined with 4D imaging data, it makes it possible to estimate the deformations of different regions of the cardiovascular structures during the cardiac cycle semiautomatically [18–21], thus overcoming the limitations of conventional, simple diameter measurements by operators.

In this study, we use 4DCT imaging data of patients undergoing TPVR following surgical repair of CHD to extract 3D geometries at end diastole and systole and compute the strain field through a DSM framework [16]. We aim to assess the local circumferential and axial RVOT/PA strains, and their ratio, and quantify the regional averages across the population to inform new device design.

## Materials and Methods

### Patient Population and 4D CT image Processing

Contrast-enhanced, retrospectively ECG-gated cardiovascular 4DCT images (Siemens Dual Source CT, Ehrlangen, Germany) were retrospectively collected from a database of CHD patients, imaged between 2019 and 2022, with surgically repaired RVOT/PAs, and scheduled for a TPVR procedure at Children’s Hospital Colorado, Denver, CO, USA. The number of phases per cardiac cycle ranged between 10 and 21. The phases captured the automatically identified left ventricle peak systole and peak diastolic phases and with a buffer of a variable number of additional phases before and after to allow for differences in right and left ventricular contraction timing. Demographics and surgical history were collected for each patient including primary diagnosis (tetralogy of Fallot, pulmonary stenosis or other), RVOT/PA repair (transannular patch, homograft, valvuloplasty, or valvotomy) and RVOT/PA dysfunction (regurgitant, mixed, or stenotic). ‘Mixed’ referred to patients with both regurgitant and stenotic RVOT/PA dysfunction as defined by cardiac magnetic resonance. Exclusion criteria were the presence of main or branch PA stents, insufficient image quality, incomplete clinical history. Institutional ethical approval for the retrospective use of the CT image data was obtained.

3D Slicer (v5.2.2 https://www.slicer.org/) medical image software was used by a single operator with 5-year experience to segment the RVOT/PA region of interest at end diastole (RVOT/PA_D_) and end systole (RVOT/PA_S_) defined by the maximum and minimum RV volume, respectively. These phases, considered close to the overall least and most loaded RVOT/PA in the axial direction during the cardiac cycle [10], were selected to demonstrate the methodology. The segmentation output was exported as a surface mesh into VMTK (Vascular Modeling Toolkit, Orobix, Bergamo, Italy) and Meshmixer (AutoDesk Inc., San Francisco, USA) for surface pre-processing. Following a previously published protocol [22], the surfaces were clipped orthogonal to the vessel centerline, proximally by the plane which retained a continuous ring of contractile myocardium, and distally by a plane at the first bifurcation of the branch PAs. The population of shapes were then rigidly registered according to the same, previously published protocols.

**Fig. 1:**
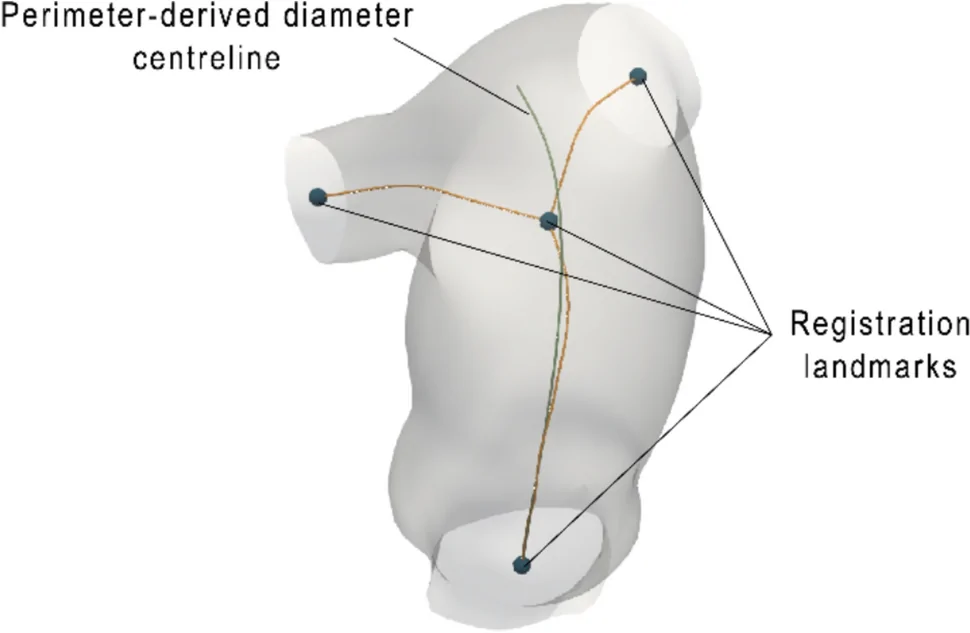
Example of patient RVOT/PA processed surface including bifurcating centerline and corresponding landmark locations and single line from inlet to bifurcation saddle for perimeter-derived diameter calculation

Landmarks (4 black dots in Fig. 1) were extracted at inlet, outlet and left PA and right PA centerline bifurcation point, and geometric measurements of surface area (SA), average perimeter-derived diameters of the RVOT/PA (D_AVE_) and RVOT/PA centerline length (L_RVOT/PA_) were computed as previously described [22].

All surfaces were re-meshed to a target edge length of 2 mm, calculated according to the sensitivity analysis method established by Bruse et al. [23]. Errors introduced by remeshing were assessed on the median of the average distance between the remeshed surfaces versus the segmented surfaces, and compared to the mean voxel size of the 4D CT images.

The RVOT/PA_D_ which had SA closest to the mean SA for the RVOT/PA_D_ population was used as the initial reference geometry for registering the population of RVOT/PA_D_ surfaces [23] based on the landmarks and the centerlines of each surface (Fig. 1) and a least-square minimisation approach. Each RVOT/PA_S_ was similarly rigidly registered to its corresponding RVOT/PA_D_ to simplify DSM computation by removing rigid body motion.

### Deformable Shape Modeling

**Fig. 2:**
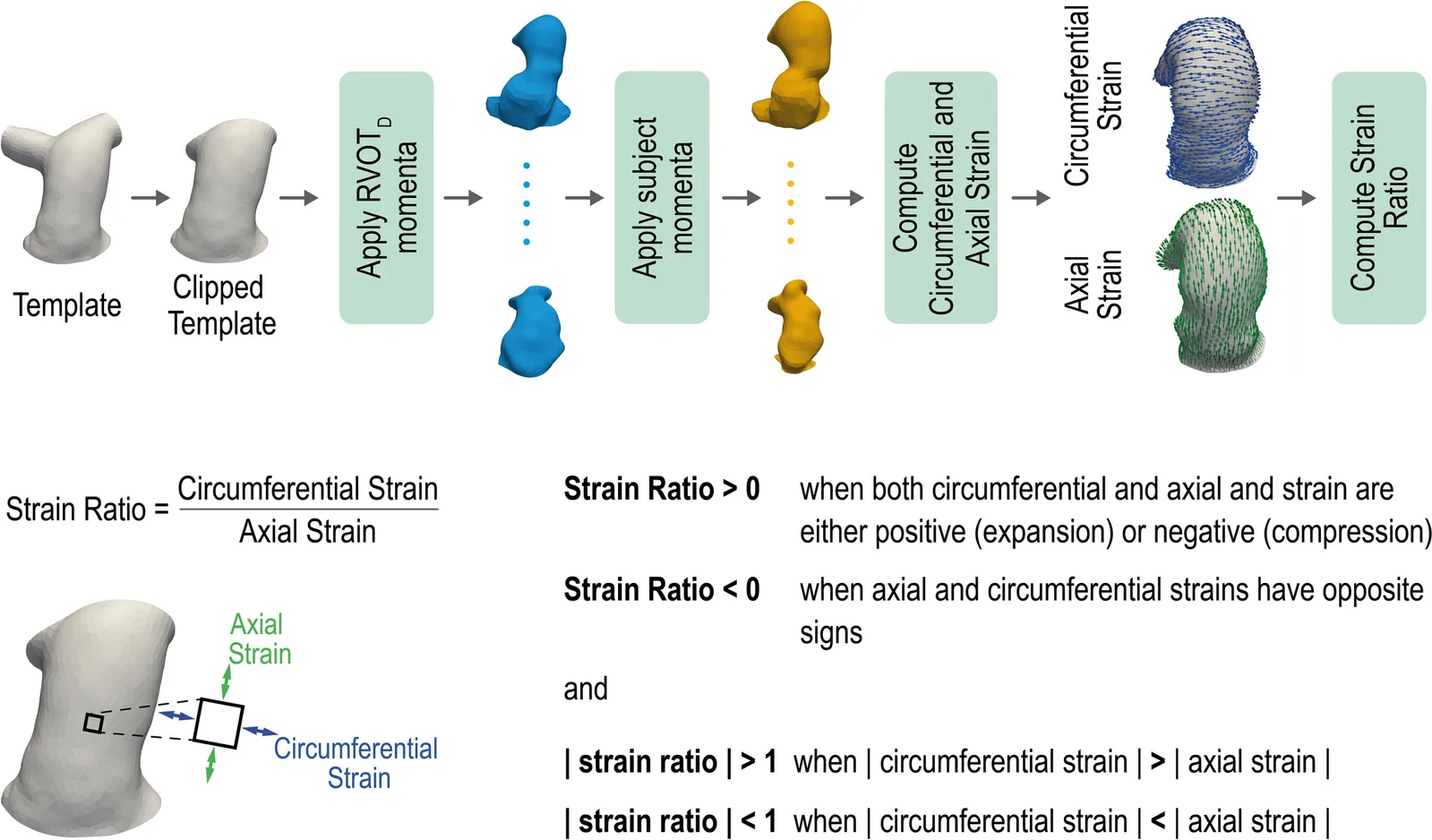
Flow Chart of the deformable shape modeling methodology which obtains point-correspondence between the clipped template. the RVOT/PA_D_ and RVOT/PA_S_ shape in order to compute axial and circumferential strain, and the proposed strain ratio. A guide on interpreting the strain ratio is included

The complete shape modeling process described below is summarized in the flow chart of Fig. 2 and used the non-parametric, non-landmark based Deformetrica package (www.deformetrica.org) [15, 16]. The Deformetrica framework deforms the reference to target shapes such that a distribution of shape features which are detected in the population are matched to achieve point-correspondence. The framework by Bruse et al. was followed to validate each step of the DSM pipeline, including kernel width optimization, population registration validation, and template validation [23]. Due to the large variation between the subjects, all computed shape models used a stepped, multiscale approach to progressively deform the template toward the target shape and avoid the introduction of reconstruction anomalies. In the first step, large kernel width and low control point density were used to marginally deform the template toward the population. These results initialized the momenta of a new computation with stepped-down kernel widths and higher control point density, repeated twice.

An RVOT/PA_D_ DSM established point-correspondence between a common template and the population of RVOT/PA_D_ surfaces [23]. The SA, L_RVOT/PA_, and D_ave_ of the template were compared to the average value of these parameters in the RVOT/PA_D_ population for template validation. The template was clipped at the bifurcation of the branch PAs (clipped template) to constrain the strain analysis to the possible implantation site of TPVR devices. In individual cases where the RVOT/PA_D_ surface was found to be much smaller than the surface of the template, the template was non-rigidly registered to the RVOT/PA_D_ surface to reduce the size difference, and thus the complexity of computing the momenta for this case.

A second DSM computed the deformations from each subject RVOT/PA_D_ to its respective RVOT/PA_S_ surface. The momenta for each RVOT/PA_D_ deformation to its registered RVOT/PA_S_ counterpart were computed on the same control point grid as the RVOT/PA_D_ DSM. The deformations of the RVOT/PA_D_ DSM were applied to the clipped template, following which the deformations of the RVOT/PA_S_ DSM were applied. In dong so, a population of clipped RVOT/PA_D_ and RVOT/PA_S_ surfaces, all with nodal correspondence between each other and with the clipped template were generated. DSM reconstruction accuracy was assessed on the median of the average distance between the surface points of the DSM reconstructed population versus those of the input population. Visual checks were performed on each patient to ensure that the propagation of each diastolic mesh to the respective systolic surface remained anatomically correct.

### Strain and Strain Ratio Estimation

The normal, axial, and circumferential vectors on each cell of the reconstructed clipped RVOT/PA_D_ surfaces were computed. Each element’s unit axial vector was defined by the tangent of the nearest point on the perimeter-derived radii centerline (Fig. 1), projected onto the element plane. The circumferential unit vector was defined by the cross-product between the axial and outward-pointing normal unit vectors. The RVOT/PA_D_ phase of each patient was set as the reference shape [19] and the Green–Lagrange strain tensor *E* calculated on each cell element (1) using the systolic displacement vectors between the clipped RVOT/PA_D_ and RVOT/PA_S_.

1 $${\bf E} = \frac{1}{2}\left( {{\bf F}^{T} {\bf F} - {\bf I}} \right)$$

where *I* = identity matrix and *F* = deformation gradient computed following the procedure described by Aggarwal et al. [19]. The resulting strain tensor was rebased to assess the circumferential, axial, and shear (resulting from torsion) strains on each subject.

The strain ratio was calculated as the ratio between the circumferential and axial strain components. The cell medians of these parameters across the population were plotted on the clipped template.

**Fig. 3:**
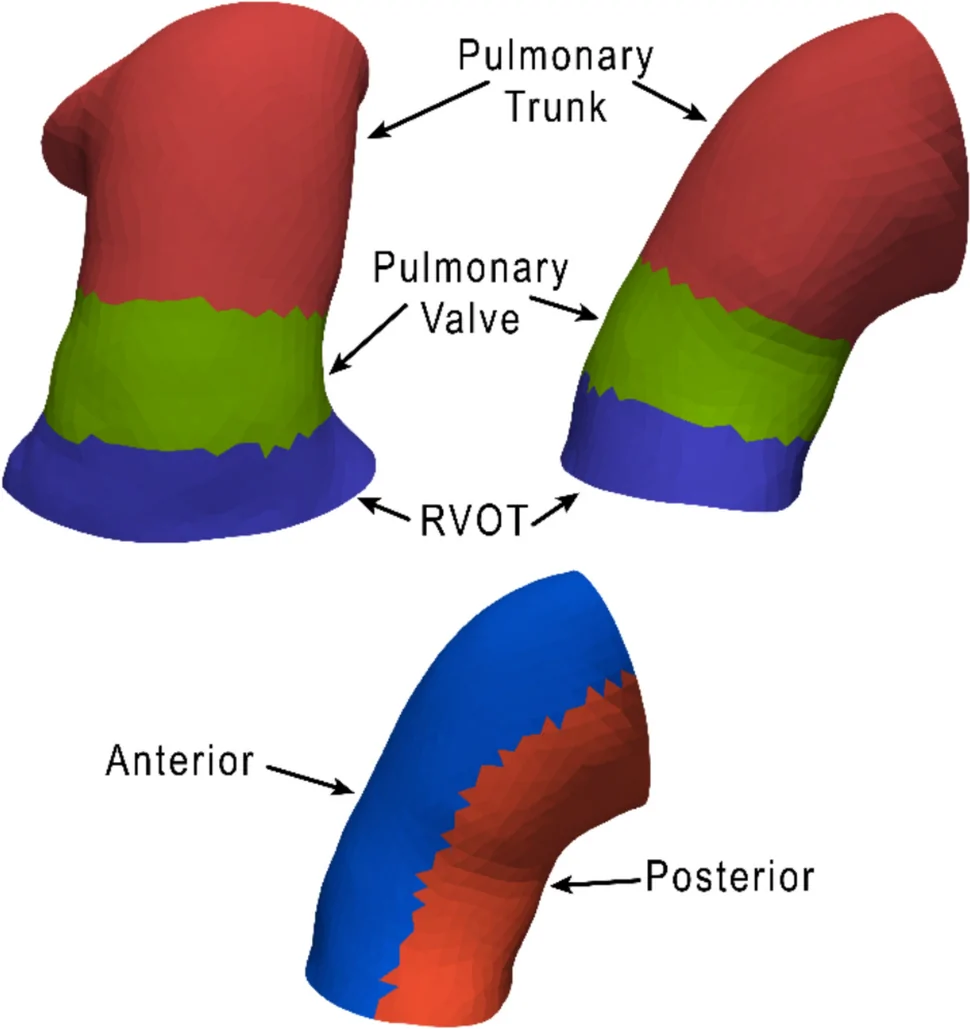
Key regions for extracting strain properties were defined as the RVOT/PA, pulmonary valve and pulmonary trunk as well as the anterior and posterior regions

The regions of the RVOT, pulmonary valve, and pulmonary trunk, as well the posterior and anterior regions shown in Fig. 3, were identified on the clipped template using VMTK, by manually selecting the mesh elements of the valve sinuses evident in the geometry, and by selecting the elements of the anterior half from the sagittal views, respectively. Leveraging point-correspondence, these regions could be propagated from the clipped template throughout the population of RVOT/PA_D_; iterative adjustments were made to ensure that the regions remained visually true when propagated to each patient and to each systole phase. The area-weighted average of the median circumferential and axial strains, and strain ratio fields on the clipped template was computed for each region.

## Results

### Patient Population and 4D CT Images

**Fig. 4:**
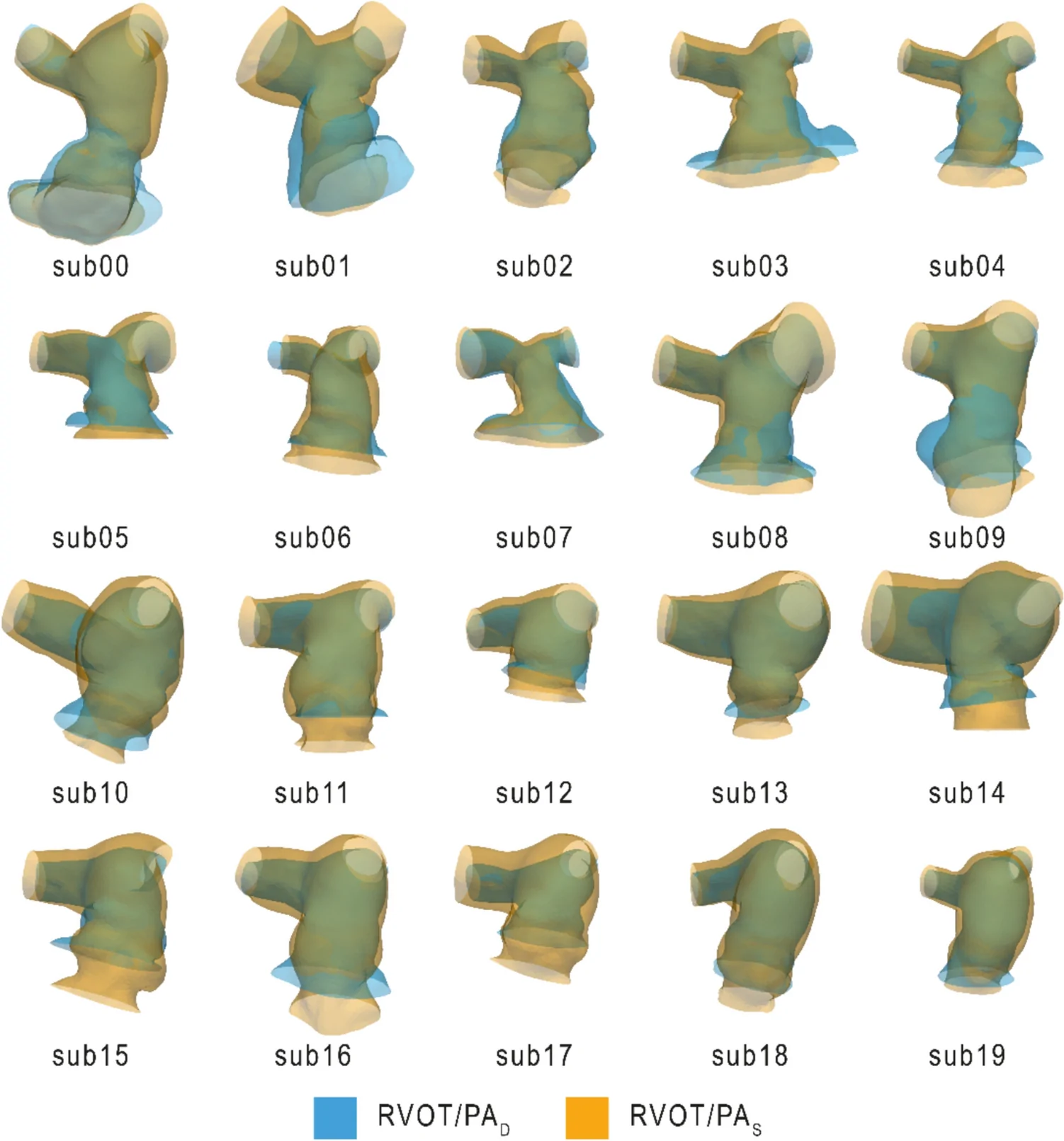
Population of RVOT/PA shapes showing each subject RVOT/PA_D_ and RVOT/PA_S_ after registration in blue and orange, respectively. Subject 00 is the homograft RVOT. Subjects 01–09 are patch RVOTs. Subjects 10–14 are valvotomy and 15–19 are valvuloplasty cases, all ordered by ascending age

From 30 patients initially selected from the TPVR clinical database, 10 were excluded from the study because of main PA or branch PA stents (*n* = 7), poor image quality (*n* = 1), and incomplete clinical data (*n* = 2). The characteristics of the remaining subjects (*n* = 20, 35% female, age at scan = 32 ± 16 years, BSA = 1.85 ± 0.31) are shown in Table 1. All patients underwent TPVR except one (Fig. 4, subject 14), who was ultimately referred for surgical valve replacement. The dominant primary diagnosis in the cohort was pulmonary stenosis (50%). All other patients were classified as tetralogy of Fallot except for one patient who had an undefined original RVOT abnormality. Surgical repairs most commonly involved a transannular patch (45%), followed by valvuloplasty and valvotomies (25% each). The cohort consists predominantly of patients with moderate to severe pulmonary regurgitation (85% of patients). No purely stenotic cases were included. One case, who was not stenotic but went for TPVR, had no regurgitant fraction on record.

**Table 1:** Demographic description of the cohort

Group	*n* = 20	Group	*n* = 20
*Diagnosis*		*RVOT/PA dysfunction*	
Pulmonary Stenosis	10 (50%)	Regurgitant	17 (85%)
ToF	9 (45%)	Mixed	2 (10%)
Other*	1 (5%)	Unknown	1 (5%)
*RVOT/PA Type*			
Homograft	1 (5%)		
Patch	9 (45%)		
Valvotomy	5 (25%)		
Valvuloplasty	5 (25%)		

ToF = Tetralogy of Fallot. Other* refers to a case that had severe homograft stenosis following a Ross procedure. The case which had unknown RVOT/PA dysfunction had no recorded regurgitation fraction but as this was not relevant for the study, remained included

The average voxel size of the CT datasets was 0.54 × 0.54 × 0.51 ± 0.2 mm. Figure 4 shows the population of RVOT/PA_D_ (blue) and RVOT/PA_S_ (yellow) shapes, superimposed on each other, following all surface processing and registration steps. The mean surface distance after remeshing versus the initial segmentation across the population of surfaces was 0.05 ± 0.10 mm – an order of magnitude smaller than the average CT voxel size in the cohort. The average geometric measurements of the RVOT/PA_D_ and RVOT/PA_S_ surfaces are summarized in Table 2, showing an increase at systole of 11% in diameter and 26% in length.

**Table 2:** Description of average geometric measurements for RVOT/PA_D_ and RVOT/PA_S_

Metric	RVOT/PA_D_	RVOT/PA_S_	Difference
Surface Area $[\mathrm{x}1{0}^{3}\text{ m}{\mathrm{m}}^{2}]$	10 ± 3	13 ± 3	3 ± 1 (29 ± 16%)
Volume $[\mathrm{x}1{0}^{3}\text{ m}{\mathrm{m}}^{3}]$	75 ± 32	108 ± 42	33 ± 20 (49 ± 28%)
Average RVOT/PA Diameter [$\mathrm{mm}]$	36 ± 7	39 ± 5	4 ± 3 (11 ± 8%)
RVOT/PA Length $[\mathrm{mm}]$	60 ± 12	75 ± 13	15 ± 7 (26 ± 14%)

### Statistical Shape Model

**Fig. 5:**
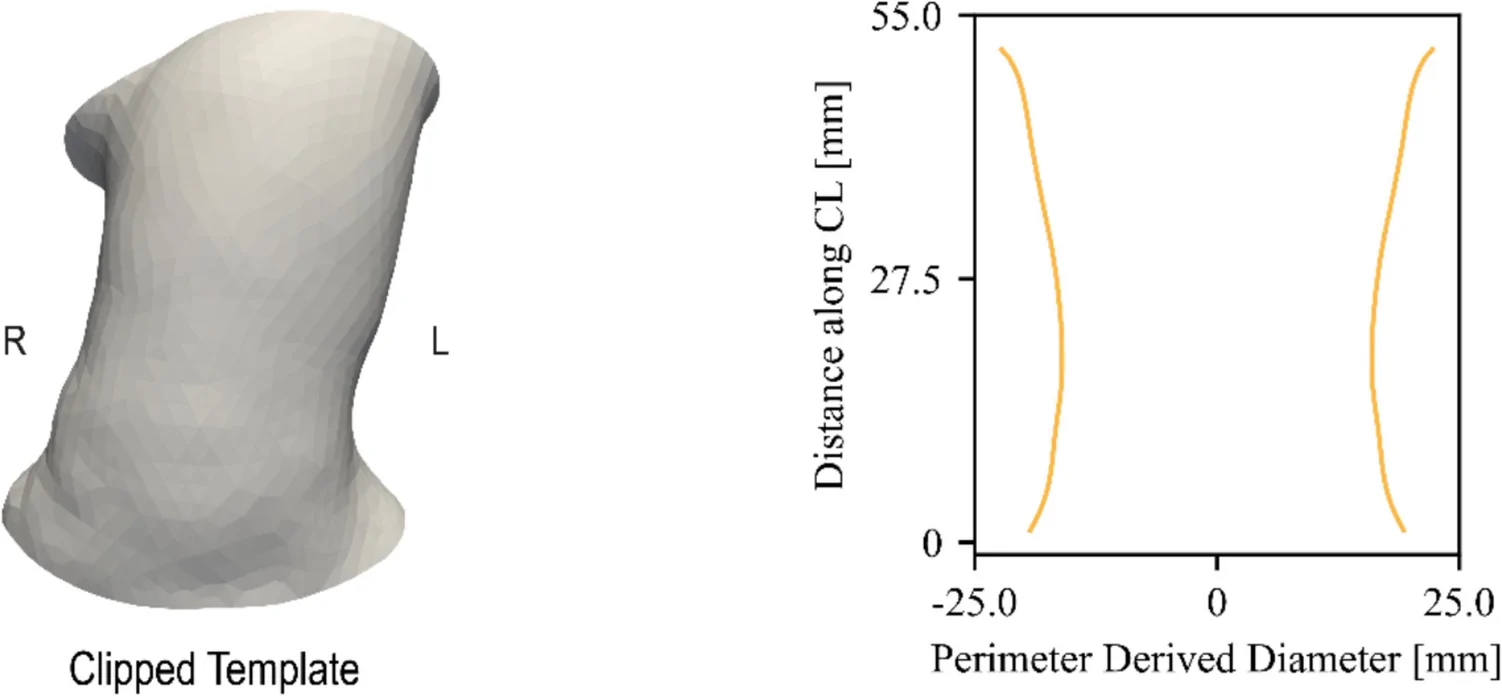
Final clipped SSM template and perimeter-derived diameter plot from the inlet to the bifurcation

The final clipped template, and the derived perimeter plot are shown in Fig. 5. The SA, V, L_RVOT/PA_, and D_AVE_ of the unclipped template, were 10 × 10^3^ mm^2^, 76 × 10^3^ mm^3^, 66 mm, and 36 mm, equivalent to 1%, 2%, 9%, and 0.5% difference from the means of the RVOT/PA_D_ surfaces (Table 2). The DSM reconstructions of both RVOT/PA_D_ and RVOT/PA_S_ surfaces had a mean surface distance from their input shapes of 0.009 ± 0.005 mm, considerably smaller than the CT images’ voxel size, and, therefore, considered acceptably accurate.

### Strains and Strain Ratio Estimation

**Fig. 6:**
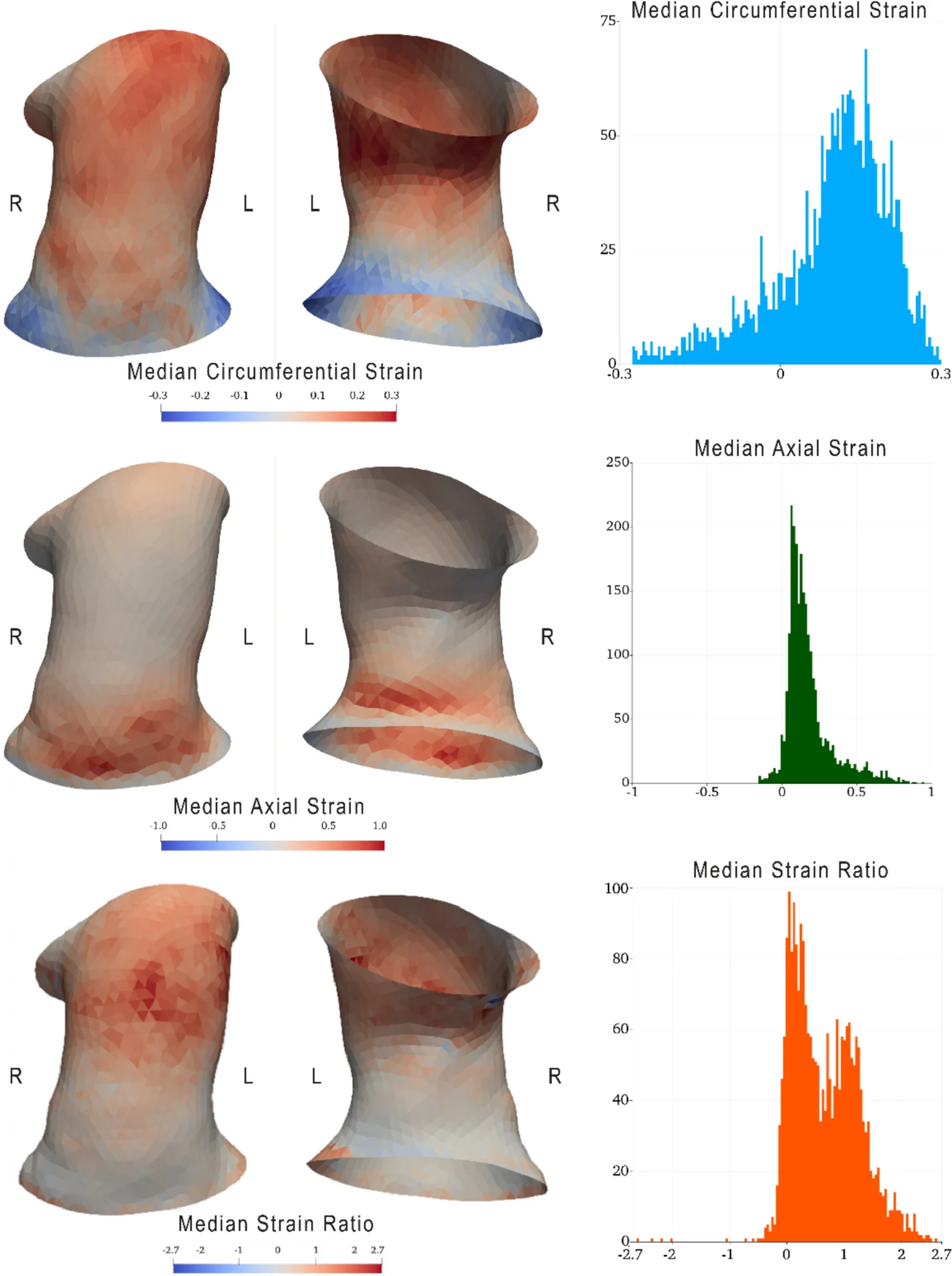
Computed circumferential and axial strains, and strain ratio as median across the population and plotted on the clipped template. Histograms show that most of the surface strains are quite small and similarly signed and, as a result, the strain ratio is positive in the majority of the vessel

The population circumferential and axial components of the Green–Lagrange strain and the strain ratio are shown in Fig. 6 on the template surface. The area-weighted average, with minimum and maximum, across the entire template surface for the median circumferential strain, axial strain, and strain ratio was 0.09 [− 0.28–0.30], 0.18 [− 0.16–0.95], and 0.66 [− 2.6–2.7], respectively. Shear resulting from torsion about the axial direction was found to be negligeable compared to the strains in the other directions, and hence the results were not included. The distributions of the circumferential and axial strains both show some negative values in the proximal ring region indicating compression of those elements in both directions. Elsewhere shows generally positive circumferential and axial strain values indicating elongation and expansion, respectively.

**Fig. 7:**
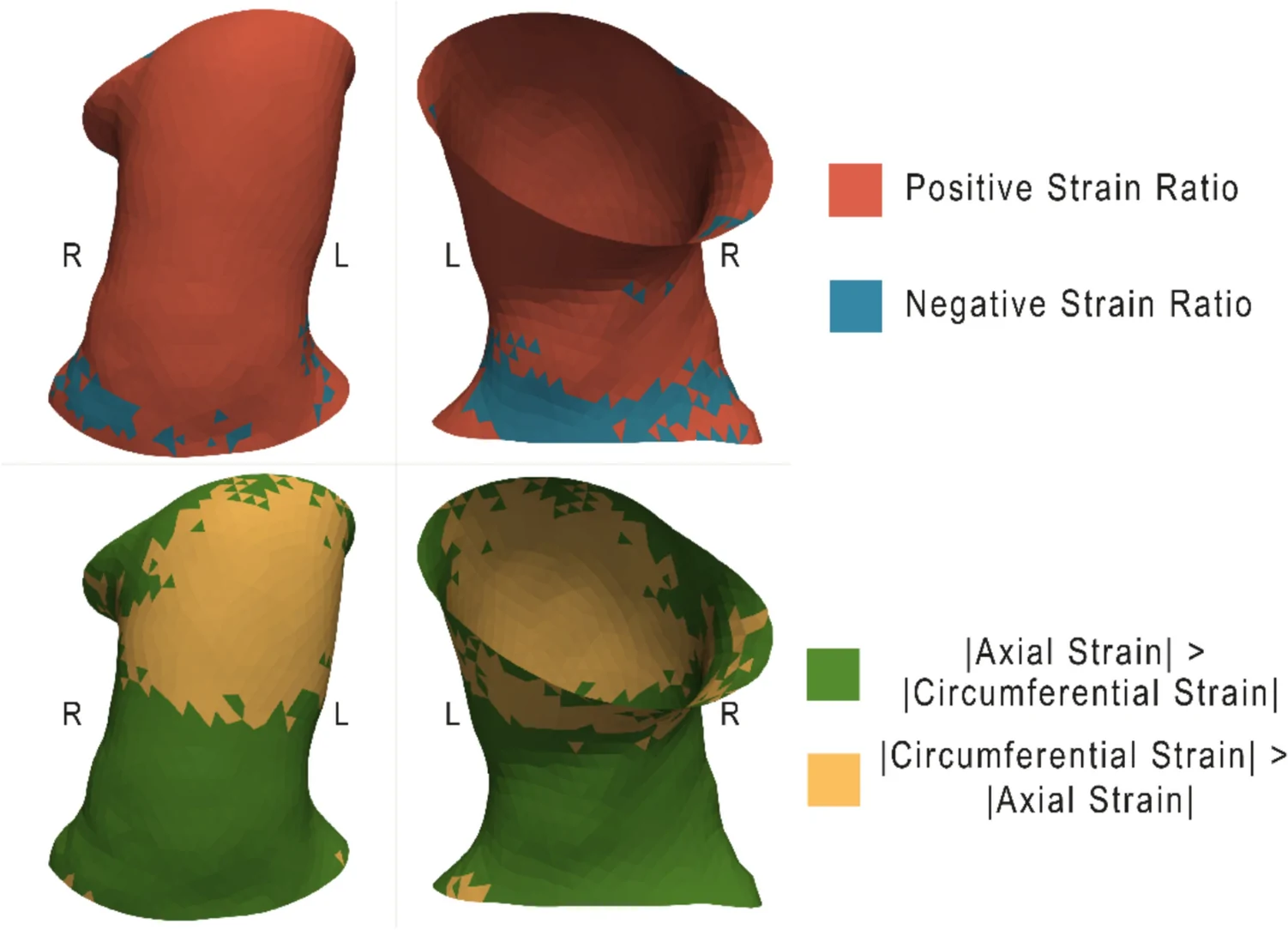
Top: Regions of positive (red) and negative (blue) median strain ratio show that 90% of the surface experiences positive strain ratios. Bottom: Regions where the magnitude of axial strain is greater than the magnitude circumferential strain (green) and vice versa (yellow). 68% of the surface, predominantly in the proximal RVOT/PA, has axial strain magnitudes greater than the circumferential strain magnitude

Figure 7 shows that the configuration of strains leads to a positive strain ratio across 90% of the template surface and axial strain greater than circumferential strain across 68% of the surface (i.e., with strain ratio between −1 and 1). The region which had negative median strain ratio, was predominantly in the proximal, posterior portion and in some cells on the anterior side, highlighted in blue in Fig. 7. Axial strains were predominantly larger than the circumferential strains in the proximal part of the RVOT/PA.

**Fig. 8:**
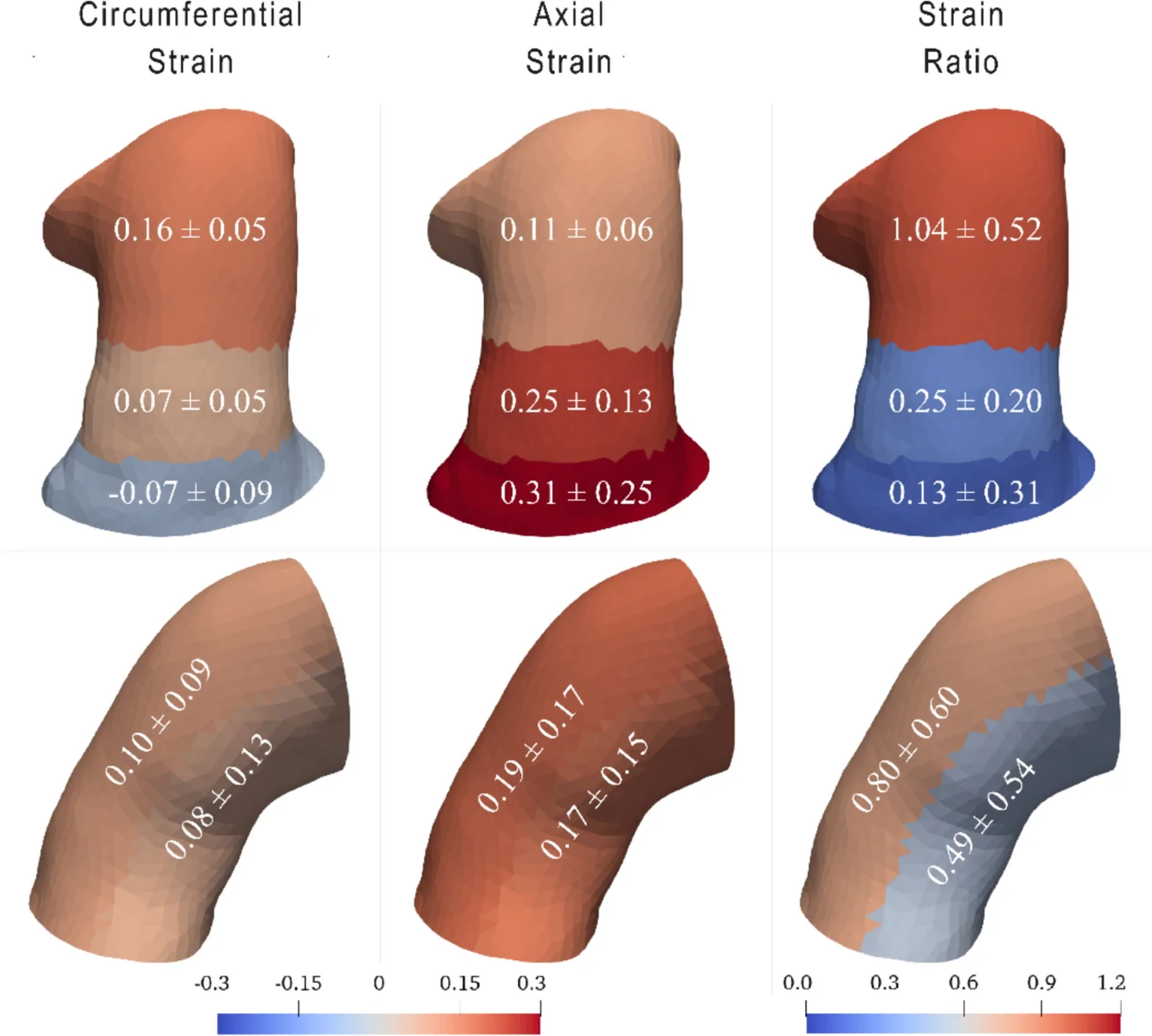
Color maps of the circumferential and axial strains and strain ratio in the defined regions of the RVOT/PA. Regional area-weighted average values, with the standard deviation are superimposed. The circumferential strain tends to increase, axial strain decrease, and strain ratio increase, progressively, from the RVOT to the pulmonary trunk region. The anterior region experiences higher strains and strain ratio than the posterior region, albeit marginal in the case of the circumferential and axial strain components

The area-weighted averages of the median circumferential and axial strain and strain ratios in the RVOT, pulmonary valve, pulmonary trunk, posterior and anterior regions are visualized and recorded in Fig. 8. The circumferential strains, when averaged over the elements of each region, were slightly negative in the proximal ring and progressively increased in the distal regions. In contrast the area-weighted average of the median axial strain was the highest in the RVOT and progressively reduced (remaining positive) when moving distally toward the PA bifurcation. The area-weighted average of the median strain ratio was positive in every region, increasingly so when transitioning from the proximal to the distal region. The anterior and posterior regions experienced similar strains in both the circumferential and axial directions, albeit slightly higher in the posterior region. These resulted in a larger difference in regional strain ratio between the posterior – more positive – and the anterior region.

## Discussion

In this study, we combined 4DCT image data and a DSM framework to compute the strains of the RVOT/PA during the cardiac cycle in a population of 20 patients undergoing TPVR. The DSM allowed quantification of the average distributions of circumferential strains, axial strains, and strain ratio across the population along the RVOT/PA. Overall circumferential strains, due to the diameter increase during systole, are considerably smaller (half) than axial strains required to accommodate the stroke volume. While strain ratios were predominantly positive – indicating that the RVOT/PA simultaneously dilates and elongates in systole – some regional differences were noted: the proximal RVOT presented the largest elongation (positive axial strains) together with some compression (slightly negative circumferential strains). The elongation in systole decreased along the pulmonary valve and pulmonary trunk, while these regions simultaneously dilated, with the distal region being the most dilated in systole. This led to an increased strain ratio along the RVOT, slightly larger than 1 in the pulmonary trunk. The proposed analysis can improve our understanding of the RVOT/PA loading conditions to support new TPVR device designs, as manufacturers could provide solutions that account for these complex dynamics.

The patients for the presented study were selected based on their need for a new pulmonary valve, with the cohort including predominantly patched and native RVOTs after valvotomy/valvuloplasty repairs. These patients present with a wide range of anatomies [2], highly dilated, often resulting in severe regurgitation, and also exhibit large dynamics [2, 9]. Indeed, the cohort overall RVOT/PA volume increased by 49% during systole (surface area by 29%) due to a simultaneous increase of 11% in RVOT/PA diameter and 26% in length. This average magnitude of length change in the population is similar to what has been previously found in the normal population [10] but with a much larger spread. However, measures of volumes, diameters, and lengths, do not provide local or regional RVOT/PA surface strain information and differences among patients. Additionally, routine clinical measurements of the RVOT/PA from CT scans [10, 24], especially in terms of diameters, are prone to errors as sensitive to operators’ choices on the cross-sectional planes. Thus, the proposed DSM, with no need for operator dependent measures and anatomical landmark selection, difficult in the context of repaired CHD, was developed to capture automatically the RVOT/PA complex surface deformations that happen in this group of patients.

Our group has previously shown the importance of 4D imaging for examining RVOT/PA geometry changes during the cardiac cycle to assess device implantation parameters [9]. 4DCT has become the standard imaging modality for patient screening for the next generation of TPVR devices, particularly through derived perimeter plots. These and similar methods used to quantify strain in the great arteries [10, 25], provide limited information, discarding most of the 4D details contained in the images. DSMs, instead, may provide an alternative measure to capture the entire 4D information for more comprehensive clinical screening. For example, in this study, we highlight the regional variations of circumferential strains, largest in the distal and anterior regions of the RVOT/PA. These regions of high circumferential strain, particularly in the distal region, may indicate areas at risk of poor device apposition or device migration. This is particularly important in cases where the TPVR device is deployed more distally than normal. On the other hand, regional differences in the axial strains, largest in the proximal and anterior regions of the RVOT/PA, are considerable and present additional load on the TPVR stent in this region. Furthermore, the median axial strain maps show reduced axial and circumferential contraction in the proximal, anterior region which may indicate the overall effect of passively deformed patch tissue.

Furthermore, the DSM methodology presented here is applicable to other 4D imaging methods such as modern 4D cine magnetic resonance imaging. This is important as centers seek avenues that reduce exposure of patients to ionizing radiation. With further investigations on larger cohorts, development of DSM methods that can be implemented in the clinical workflow, and the growing market of new available TPVR devices, these models may be able to support better patient screening and device selection for optimal outcomes.

4DCT in these patients was performed for the purpose of screening for newer self-expanding valve platforms (Harmony, Medtronic, Minneapolis MN; Alterra, Edwards Life Sciences, Irvine, CA; Venus P-Valve; Venus Medtech, Hang Zhou, China). These are designed for native RVOT/PAs and are based on diamond-like cell shapes and circular cross-sections of varying diameters along the length [4]. These designs force the devices to shorten during dilation, in disagreement with the natural dynamics of the unconstrained RVOT/PA identified in this study. Hence, TPVR results in stiffening of the RVOT/PA not only in the radial direction, as already widely reported, but, mostly in the axial direction. By altering the afterload compliance, this may, on one hand, affect the ability of the RVOT/PA to accommodate the cardiac stroke volume, thus impacting on the overall hemodynamics performance. On the other hand, axial stiffening of the RVOT/PA may transfer the cyclic axial loads to the TPVR stent, thus increasing the risk of fatigue failure and reducing the lifespan of the device [26]. More natural RVOT/PA deformations could be achieved considering new auxetic TPVR device designs [27, 28] that allow for more flexible solutions with greater axial elongation when dilating and asymmetric designs that can accommodate strain ratio regional differences, as indicated in this study.

Furthermore, not only the designs of the TPVR devices, but also the standards for assessment of their mechanical and long-term performance should consider more realistic loading conditions. Stent mechanical performance testing focuses, among others, on radial stiffness and ability of the device to withstand cyclic fatigue based on hydrodynamic pulsatile loading conditions that replicate physiologically relevant diameter changes. However, no tests are purposely carried out to assess the mechanical behavior when the device is loaded in the axial direction. Current standard tests, that may be suitable for other stent applications or transcatheter aortic valve implantation (TAVI) devices – the aortic root in TAVI patients is often stiff and calcified – do not account for the complex range of in vivo loading conditions encountered in TPVR patients.

As the database of 4D scans increases with more patients screened for TPVR, there is a growing opportunity to expand image post-processing methods such as DSMs to study in situ RVOT/PA dynamics. This would benefit future TPVR device designs and may provide further clinical insight for TPVR patient selection criteria.

### Limitations

This study includes a small sample of patients with different diagnosis and RVOT/PA repair types. The size of the cohort and number of patients in each sub-group of repair type do not carry sufficient power to characterize completely the population or its sub-groups. The results hence present evidence to support commonly observed dynamics and motivate a larger study to find differences between sub-groups of RVOT/PA repairs.

Segmentation model processing for DSM construction included steps for smoothing and mesh refinement of the segmented surfaces. Errors due to these operations and created by the DSM itself were smaller than the CT images’ voxel size, and, therefore, considered acceptable.

Our proposed methodology computed the strains between two time points over the cardiac cycle (end of systole and end diastole). These strains do not necessarily provide insight into the maximum axial or circumferential strains over the cardiac cycle, due to regional and individual variations [9]. Extending the image analysis to include multiple phases may uncover higher strains in RVOT/PA loading, more relevant for TPVR.

Additionally, the computed strains are not absolute, but rather relative to the end-diastolic phase, which was chosen as reference, as the developed model does not take into account the pre-strains experienced by the RVOT/PAs at diastole. Computing the pre-strain state requires complex inverse modeling approaches with several material/loading condition assumptions, and was outside the scope of this study. Hence the computed strains should be considered as relative and not a representation of the total biomechanical state of the RVOT/PA, limiting considerations on the overall tissue biomechanical response.

Strains in the RVOT region show predominant axial elongation which has been previously described [29]. Furthermore, analyzing population and region averaged strain measurements limits the ability to asses the impact of the presence of a patch, adhesions or sutures on local, patient-specific, strains. The results presented here are intended to be population level characteristics and focus on the fact that prominent axial strains exist in the population.

## Conclusion

In this study our DSM computed the local deformations, axial and circumferential strains of the RVOT/PA during systole in a cohort of patients who were assessed for TPVR late after surgical CHD repair. This method, applied for the first time to the RVOT/PA, requires little supervision to establish point-correspondence and may be applied to other 4D imaging modalities. We also propose the ‘strain ratio’ metric as a compact way of quantifying the relationship between axial and circumferential strain. From the population average of these metrics, we identify that the RVOT/PA in this population generally undergoes significant elongation during systole as compared to dilation. The strain ratio remains largely positive indicating co-expansion or co-contraction in the axial and circumferential directions and confirms clinical observation. The results presented here may impact future device designs and patient selection for TPVR.

## Data Availability

The raw data supporting the conclusions of this article will be made available by the authors on request due to privacy and ethical reasons.
